# 1,3-Bis(2-thienylmeth­yl)-4,5-dihydro­imidazolium trichlorido(η^6^-*p*-cymene)ruthenate(II)

**DOI:** 10.1107/S1600536809000464

**Published:** 2009-01-10

**Authors:** Hakan Arslan, Don VanDerveer, İsmail Özdemir, Nevin Gürbüz, Yetkin Gök, Bekir Çetinkaya

**Affiliations:** a Department of Natural Sciences, Fayetteville State University, Fayetteville, NC 28301, USA; b Department of Chemistry, Faculty of Pharmacy, Mersin University, Mersin, TR 33169, Turkey; c Department of Chemistry, Clemson University, Clemson, SC 29634, USA; d Department of Chemistry, Faculty of Science and Arts, İnönü University, Malatya, TR 44280, Turkey; e Department of Chemistry, Faculty of Science, Ege University, Bornova-İzmir, TR 35100, Turkey

## Abstract

The asymmetric unit of the title compound, (C_13_H_15_N_2_S_2_)[RuCl_3_(C_10_H_14_)], contains a 1,3-(2-thienylmeth­yl)-4,5-dihydro­imidazolium cation and a trichlorido(*η* ^6^-*p*-cymene)ruthenate(II) anion. The thio­phene rings of the cation are disordered by an 180° rotation about the thio­phene–CH_2_ bonds with occupancies of 0.847 (5)/0.153 (5) and 0.700 (5)/0.300 (5), respectively. The Ru atom exhibits a distorted octa­hedral coordination with the benzene ring of the *p*-cymene ligand formally occupying three sites and three Cl atoms occupying the other three sites. The short C—N bond lengths in the imidazoline ring indicate partial electron delocalization within the N—C—N fragment. Cation and anions are connected through five inter­molecular C—H⋯Cl hydrogen bonds and one C—H⋯π hydrogen bond, forming a three-dimensional hydrogen-bonded network.

## Related literature

For the synthesis, see: Yaşar *et al.* (2008 ▶). Özdemir *et al.* (2008 ▶, 2007 ▶, 2005 ▶). For general background, see: Herrmann *et al.* (1995 ▶); Herrmann (2002 ▶); Arduengo & Krafczyc (1998 ▶). For related compounds, see: Arslan *et al.* (2007 ▶, 2005*a* ▶,*b* ▶) and references therein; Sonar *et al.* (2004 ▶, 2005*a* ▶,*b* ▶); Wagner *et al.* (2006*a* ▶,*b* ▶); Crundwell *et al.* (2002 ▶); Linehan *et al.* (2003 ▶); Liu *et al.* (2004 ▶); Navarro *et al.* (2006 ▶); Therrien *et al.* (2004 ▶). For bond-length data, see: Allen *et al.* (1987 ▶).
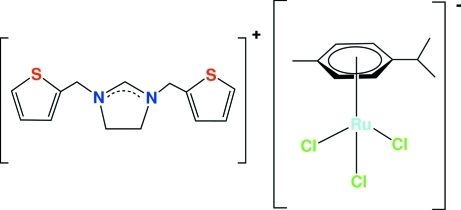


## Experimental

### 

#### Crystal data

(C_13_H_15_N_2_S_2_)[RuCl_3_(C_10_H_14_)]
*M* *_r_* = 605.02
Triclinic, 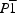

*a* = 9.910 (2) Å
*b* = 11.600 (2) Å
*c* = 12.659 (3) Å
α = 84.95 (3)°
β = 67.05 (3)°
γ = 74.14 (3)°
*V* = 1288.8 (6) Å^3^
*Z* = 2
Mo *K*α radiation
μ = 1.10 mm^−1^
*T* = 153 (2) K
0.48 × 0.19 × 0.17 mm

#### Data collection

Rigaku AFC-8S Mercury CCD diffractometer
Absorption correction: multi-scan (*REQUAB*; Jacobson, 1998 ▶) *T*_min_ = 0.621, *T*_max_ = 0.836
11095 measured reflections
4557 independent reflections
4062 reflections with *I* > 2σ(*I*)
*R*_int_ = 0.019

#### Refinement

*R*[*F* ^2^ > 2σ(*F* ^2^)] = 0.032
*wR*(*F* ^2^) = 0.084
*S* = 1.13
4557 reflections
285 parameters
H-atom parameters constrained
Δρ_max_ = 0.43 e Å^−3^
Δρ_min_ = −0.68 e Å^−3^

### 

Data collection: *CrystalClear* (Rigaku/MSC, 2006 ▶); cell refinement: *CrystalClear*; data reduction: *CrystalClear*; program(s) used to solve structure: *SHELXTL* (Sheldrick, 2008 ▶); program(s) used to refine structure: *SHELXTL*; molecular graphics: *SHELXTL*; software used to prepare material for publication: *SHELXTL*.

## Supplementary Material

Crystal structure: contains datablocks global, I. DOI: 10.1107/S1600536809000464/hg2465sup1.cif

Structure factors: contains datablocks I. DOI: 10.1107/S1600536809000464/hg2465Isup2.hkl

Additional supplementary materials:  crystallographic information; 3D view; checkCIF report

## Figures and Tables

**Table 1:** Hydrogen-bond geometry (Å, °)

*D*—H⋯*A*	*D*—H	H⋯*A*	*D*⋯*A*	*D*—H⋯*A*
C11—H11⋯Cl1^i^	0.96	2.62	3.450 (4)	144
C14—H14*A*⋯Cl1^i^	0.96	2.82	3.553 (4)	134
C19—H19*A*⋯Cl2^i^	0.96	2.81	3.671 (4)	150
C23—H23⋯Cl1^ii^	0.96	2.66	3.549 (4)	154
C14—H14*B*⋯*Cg*2^iii^	0.96	2.83	3.784 (5)	171
C19—H19*B*⋯Cl1	0.96	2.86	3.759 (5)	157

Symmetry codes: (i) 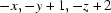; (ii) 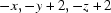; (iii) 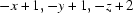. *Cg*1 is the centroid of the S2,C20–C23 thio­phene ring.

**Fig. 1.**
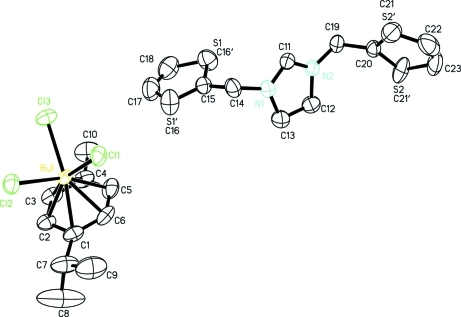
The molecular structure of the title compound, showing the atom-numbering scheme and displacement ellipsoids drawn at the 50% probability level.

**Fig. 2.**
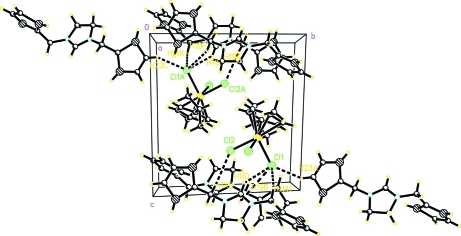
A packing diagram for (I). Symmetry: H19I, H14I, H11D, -x, 1 - y, 2 - z; H23A, -x, 2 - y, 2 - z; H23C, 1 + x, -1 + y, -1 + z; H19K, H14K, H11E, 1 + x, y,-1 + z; H19F, Cl1A, Cl2A, 1 - x,1 - y,1 - z.

**Fig. 3.**
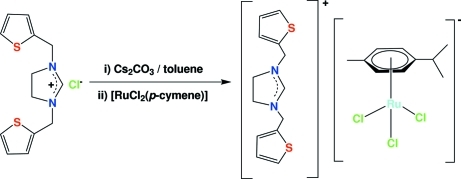
Synthesis of the title compound.

## supplementary crystallographic information

### Comment

Metal-carbene compounds, such as *N*-heterocyclic carbene palladium and ruthenium complexes, are important catalysts that have a wide range of applications such as Suzuki-Miyaura, Sonogashira, Stille and Heck reactions (Herrmann 2002; Herrmann *et al.*, 1995; Navarro *et al.*, 2006; Arduengo & Krafczyc, 1998).

In previous papers, we have described the synthesis, characterization and applications of palladium, platinum and ruthenium *N*-heterocyclic carbene complexes as catalysts (Yaşar *et al.*, 2008; Arslan *et al.*, 2007, 2005*a*, 2005*b*, and references therein; Özdemir *et al.*, 2008, 2007, 2005, and references therein). In view of these important attributes of *N*-heterocyclic carbene derivatives, we report here the crystal structure of one of them. The title compound consists 1,3-*bis*(thiophen-2-ylmethyl)-4,5-dihydro-1*H*-imidazolium cation and a trichloro(*η*^6^-*p*-cymene) ruthenium(II) anion. The molecular structure of the title compound, (I), is depicted in Fig. 1. Cation and anion groups are connected with five intermolecular C—H···Cl hydrogen bonds and one C—H···π hydrogen bond, forming a three-dimensional hydrogen-bonding network (Fig. 2).

A flip disorder of both thiophene rings in 1,3-*bis*(thiophen-2-ylmethyl)-4,5-dihydro-1*H*-imidazolium cation is observed. There are two positions of both thiophene rings, rotated by 180°.  The crystal structure of the cation contains four disordered atoms, S1, S2, C16, and C21. The site occupancy factors refined to 0.847 (5) and 0.153 (5) for the S1—C15—C16—C17—C18 ring, and 0.700 (5) and 0.300 (5) for the S2—C20—C21—C22—C23 ring. A similar thiophene ring disorder has been observed in some thiophene derivatives, such as (*Z*)-3-(1-methyl-1*H*-indol-3-yl)-2-(thiophen-3-yl)acrylonitrile (Sonar *et al.*, 2004), (*Z*)-2-(3-thienyl)-3-(3,4,5-trimethoxyphenyl)acrylonitrile (Sonar *et al.*, 2005*a*), (*Z*)-3-(1*H*-Indol-3-yl)-2-(3-thienyl)acrylonitrile and (*Z*)-3-[1-(4-*tert*-butylbenzyl)-1*H*-indol-3-yl]-2-(3-thienyl)acrylonitrile (Sonar *et al.*, 2005*b*), 1,2-di-3-thienyl-2-hydroxyethanone(3,3-thenoin) (Crundwell *et al.*, 2002), 3-[2-(anthracen-9-yl)ethenyl] thiophene, (Wagner *et al.*, 2006*a*), 2,5-bis(2-cyano-2-thienylvinyl)thiophene (Wagner *et al.*, 2006*b*), and 1,4-diphenyl-2,3-dithien-3-ylcyclopentadien-1-one (Linehan *et al.*, 2003). In addition, all thiophene rings in the cation are almost planar; the maximum deviations from the least squares planes are 0.019 (4)Å for C16 and 0.006 (6)Å for C22.

The coordination geometry of ruthenium is pseudooctahedral, with an average Ru—Cl bond distance of 2.430Å. The ruthenium atom exhibits a distorted octahedral coordination with the benzene ring of the *p*-cymene ligand formally occupying three sites and three chloride atoms occupying other three sites. The distance between the centroid of the *p*-cymene ring and ruthenium is 1.6493 (15) Å, which is longer than that reported in other ruthenium compounds (Liu *et al.*, 2004; Therrien *et al.*, 2004). All the other bond lengths in (I) are in normal ranges (Allen *et al.*, 1987).

The imidazolidine ring is almost planar, the deviations from planarity of ring are N1 0.002 (3), C11 0.001 (4), N2 0.004 (3), C12 0.005 (4), C13 0.004 (4)Å. The some C—N bond lengths (N1—C11 = 1.307 (4)Å and N2—C11 = 1.302 (4)Å) for the imidazolidine ring are shorter than the average single C—N bond length of 1.48Å, thus showing double bond character in these C—N bonds. The other CN bonds length (N1—C13 1.458 (5), N1—C14 1.462 (4), N2—C19 1.460 (4) and N2—C12 1.466 (4)Å) is agree with 1.48Å C—N single bond lengths. This information indicates a partial electron delocalization within the N1—C11—N2 fragment.

The crystal packing is shown in Fig. 2. Five intermolecular C—H···Cl hydrogen bonds link the molecules of (I) and generate a three-dimensional hydrogen bonded framework. In addition, a C14 (*x, y, z*)-H···π (S2—C20—C21—C22—C23, thiophene ring; 1 - *x*, 1 - *y*, 2 - *z*) hydrogen bond is observed in the title compound, Table 1.

### Experimental

A suspension of 1,3-*bis*(thiophen-2ylmethyl)-,4,5-dihydro-1*H*-imidazolium chloride (1.1 mmol), Cs_2_CO_3 _(1.2 mmol) and [RuCl_2_(*p*-cymene)] (0.5 mmol) was heated under reflux in degassed toluene (20 ml) for 7 h (Fig. 3). The reaction mixture was then filtered while hot, and the volume was reduced to about 10 ml before addition of *n*-hexane (15 ml). The precipitate formed was crystallized from CH_2_Cl_2_: hexane (5:10 ml) to give the complex as red-brown crystals. Yields: 0.208 g, 69%. *M*.p.: 227–228°C. ^1^H NMR(CDCl_3_) δ: 1.39 (d, 6H, *J *= 6.9 Hz, CH_3_(C_6_H_4_)CH(C*H*_3_)_2_), 2.29 (s, 3H, C*H*_3_(C_6_H_4_)CH(CH_3_)_2_), 3.21 (m, 1H, CH_3_(C_6_H_4_)C*H*(CH_3_)_2_)_, _3.79 (s, 4H, NC*H*_2_C*H*_2_N), 4.12 (s, 4H, C*H*_2_C_4_H_3_S), 5.29 and 5.58 (d, 4H, *J *= 5.8 Hz, CH_3_(C_6_*H*_4_)CH(CH_3_)_2_), 7.03–7.68 (m, 6H, C_4_*H*_3_S), 8.99 (s, 1H, 2-CH). ^13^C NMR (CDCl_3_) δ: 18.6 (CH_3_(C_6_H_4_)CH(*C*H_3_)_2_), 22.3 (*C*H_3_(C_6_H_4_)CH(CH_3_)_2_), 30.8 (CH_3_(C_6_H_4_)*C*H(CH_3_)_2_), 47.0 (N*C*H_2_*C*H_2_N), 47.1 (*C*H_2_C_4_H_3_S), 79.6, 81.8, 96.5 and 100.8 (CH_3_(*C*_6_H_4_)CH(CH_3_)_2_), 126.7, 127.5, 129.1 and 135.2 (*C*_4_H_3_S), 159.3 (2-*C*H). Anal. Calc. for C_23_H_29_S_2_N_2_RuCl_3_: C, 45.66; H, 4.83; N, 4.63%. Found: C, 45.71; H, 4.89; N, 4.69%.

### Refinement

All H atoms attached to carbons were geometrically fixed and allowed to ride on the corresponding non-H atom with C—H = 0.96 Å, and *U*_iso_(H) = 1.5*U*_eq_(C) of the attached C atom for methyl H atoms and 1.2*U*_eq_(C) for other H atoms.

### Crystal data

**Table:**

(C_13_H_15_N_2_S_2_)[RuCl_3_(C_10_H_14_)]	*Z* = 2
*M**_r_* = 605.02	*F*(000) = 616
Triclinic, *P*1	*D*_x_ = 1.559 Mg m^−^^3^
Hall symbol: -P 1	Mo *K*α radiation, λ = 0.71073 Å
*a* = 9.910 (2) Å	Cell parameters from 5195 reflections
*b* = 11.600 (2) Å	θ = 3.3–26.4°
*c* = 12.659 (3) Å	µ = 1.10 mm^−^^1^
α = 84.95 (3)°	*T* = 153 K
β = 67.05 (3)°	Rod, red
γ = 74.14 (3)°	0.48 × 0.19 × 0.17 mm
*V* = 1288.8 (6) Å^3^	

### Data collection

**Table:**

Rigaku AFC-8S Mercury CCD diffractometer	4557 independent reflections
Radiation source: Sealed Tube	4062 reflections with *I* > 2σ(*I*)
Graphite Monochromator	*R*_int_ = 0.019
Detector resolution: 14.6306 pixels mm^-1^	θ_max_ = 25.2°, θ_min_ = 3.3°
ω scans	*h* = −11→11
Absorption correction: multi-scan (Jacobson, 1998)	*k* = −11→13
*T*_min_ = 0.621, *T*_max_ = 0.836	*l* = −15→15
11095 measured reflections	

### Refinement

**Table:**

Refinement on *F*^2^	Primary atom site location: structure-invariant direct methods
Least-squares matrix: full	Secondary atom site location: difference Fourier map
*R*[*F*^2^ > 2σ(*F*^2^)] = 0.032	Hydrogen site location: inferred from neighbouring sites
*wR*(*F*^2^) = 0.084	H-atom parameters constrained
*S* = 1.13	*w* = 1/[σ^2^(*F*_o_^2^) + (0.0406*P*)^2^ + 0.9404*P*] where *P* = (*F*_o_^2^ + 2*F*_c_^2^)/3
4557 reflections	(Δ/σ)_max_ = 0.001
285 parameters	Δρ_max_ = 0.43 e Å^−^^3^
0 restraints	Δρ_min_ = −0.68 e Å^−^^3^

### Special details

**Table:**

Geometry. All e.s.d.'s (except the e.s.d. in the dihedral angle between two l.s. planes) are estimated using the full covariance matrix. The cell e.s.d.'s are taken into account individually in the estimation of e.s.d.'s in distances, angles and torsion angles; correlations between e.s.d.'s in cell parameters are only used when they are defined by crystal symmetry. An approximate (isotropic) treatment of cell e.s.d.'s is used for estimating e.s.d.'s involving l.s. planes.
Refinement. Refinement of *F*^2^ against ALL reflections. The weighted *R*-factor *wR* and goodness of fit *S* are based on *F*^2^, conventional *R*-factors *R* are based on *F*, with *F* set to zero for negative *F*^2^. The threshold expression of *F*^2^ > σ(*F*^2^) is used only for calculating *R*-factors(gt) *etc*. and is not relevant to the choice of reflections for refinement. *R*-factors based on *F*^2^ are statistically about twice as large as those based on *F*, and *R*- factors based on ALL data will be even larger.

### Fractional atomic coordinates and isotropic or equivalent isotropic displacement parameters (Å^2^)

**Table:**

	*x*	*y*	*z*	*U*_iso_*/*U*_eq_	Occ. (<1)
Ru1	−0.02777 (3)	0.69238 (2)	0.639788 (19)	0.03323 (9)	
Cl1	−0.10558 (9)	0.78990 (7)	0.82284 (6)	0.04448 (19)	
Cl2	0.08996 (10)	0.51572 (7)	0.72233 (7)	0.04740 (19)	
Cl3	−0.26047 (9)	0.62550 (8)	0.71790 (7)	0.0506 (2)	
C1	0.1893 (4)	0.7262 (3)	0.5295 (3)	0.0484 (8)	
C2	0.1573 (4)	0.6370 (3)	0.4777 (3)	0.0462 (8)	
H2	0.2310	0.5610	0.4550	0.055*	
C3	0.0226 (4)	0.6570 (4)	0.4593 (3)	0.0512 (8)	
H3	0.0058	0.5957	0.4235	0.061*	
C4	−0.0892 (4)	0.7679 (4)	0.4935 (3)	0.0534 (9)	
C5	−0.0618 (5)	0.8573 (3)	0.5467 (3)	0.0559 (9)	
H5	−0.1363	0.9327	0.5705	0.067*	
C6	0.0761 (4)	0.8346 (3)	0.5644 (3)	0.0510 (8)	
H6	0.0924	0.8953	0.6015	0.061*	
C7	0.3370 (5)	0.6951 (5)	0.5491 (4)	0.0721 (12)	
H7	0.3489	0.6158	0.5792	0.087*	
C8	0.4683 (7)	0.6885 (10)	0.4361 (5)	0.168 (4)	
H8A	0.4600	0.7659	0.4019	0.251*	
H8B	0.4671	0.6316	0.3860	0.251*	
H8C	0.5617	0.6636	0.4484	0.251*	
C9	0.3364 (6)	0.7746 (6)	0.6359 (4)	0.0889 (16)	
H9A	0.4234	0.7411	0.6554	0.133*	
H9B	0.2455	0.7811	0.7034	0.133*	
H9C	0.3398	0.8527	0.6046	0.133*	
C10	−0.2363 (5)	0.7879 (5)	0.4785 (4)	0.0864 (15)	
H10A	−0.2231	0.8116	0.4013	0.130*	
H10B	−0.3119	0.8499	0.5311	0.130*	
H10C	−0.2683	0.7151	0.4937	0.130*	
S1	0.29307 (13)	0.18189 (11)	0.86238 (11)	0.0636 (4)	0.847 (5)
C16'	0.29307 (13)	0.18189 (11)	0.86238 (11)	0.0636 (4)	0.153 (5)
H16'	0.2049	0.2487	0.8803	0.076*	0.153 (5)
S2	0.13328 (18)	0.84868 (10)	0.98665 (14)	0.0673 (5)	0.700 (5)
C21'	0.13328 (18)	0.84868 (10)	0.98665 (14)	0.0673 (5)	0.300 (5)
H21'	0.1475	0.8704	0.9088	0.081*	0.300 (5)
N1	0.4121 (3)	0.3556 (2)	0.9546 (2)	0.0456 (6)	
N2	0.2449 (3)	0.5262 (2)	0.9644 (2)	0.0396 (6)	
C11	0.2748 (3)	0.4218 (3)	1.0096 (3)	0.0374 (6)	
H11	0.2029	0.3959	1.0769	0.045*	
C12	0.3763 (4)	0.5391 (3)	0.8622 (3)	0.0561 (9)	
H12A	0.4119	0.6054	0.8711	0.067*	
H12B	0.3525	0.5501	0.7948	0.067*	
C13	0.4936 (5)	0.4194 (3)	0.8565 (3)	0.0631 (10)	
H13A	0.5238	0.3766	0.7860	0.076*	
H13B	0.5819	0.4315	0.8633	0.076*	
C14	0.4669 (4)	0.2283 (3)	0.9737 (3)	0.0473 (8)	
H14A	0.4110	0.2105	1.0515	0.057*	
H14B	0.5718	0.2112	0.9631	0.057*	
C15	0.4510 (4)	0.1486 (3)	0.8940 (3)	0.0438 (7)	
S1'	0.5553 (4)	0.0368 (3)	0.8423 (3)	0.0759 (12)	0.153 (5)
C16	0.5553 (4)	0.0368 (3)	0.8423 (3)	0.0759 (12)	0.847 (5)
H16	0.6534	0.0029	0.8456	0.091*	0.847 (5)
C17	0.4850 (6)	−0.0147 (4)	0.7846 (4)	0.0743 (12)	
H17	0.5312	−0.0919	0.7467	0.089*	
C18	0.3504 (6)	0.0533 (4)	0.7880 (4)	0.0732 (12)	
H18	0.2925	0.0307	0.7515	0.088*	
C19	0.1011 (4)	0.6183 (3)	1.0051 (3)	0.0409 (7)	
H19A	0.0249	0.5844	1.0610	0.049*	
H19B	0.0701	0.6438	0.9419	0.049*	
C20	0.1103 (3)	0.7249 (3)	1.0576 (3)	0.0407 (7)	
S2'	0.0919 (3)	0.7370 (2)	1.18444 (19)	0.0656 (8)	0.300 (5)
C21	0.0919 (3)	0.7370 (2)	1.18444 (19)	0.0656 (8)	0.700 (5)
H21	0.0762	0.6796	1.2445	0.079*	0.700 (5)
C22	0.1046 (5)	0.8587 (6)	1.1876 (5)	0.0860 (17)	
H22	0.0993	0.8925	1.2561	0.103*	
C23	0.1235 (5)	0.9205 (3)	1.0935 (5)	0.0765 (14)	
H23	0.1310	1.0017	1.0887	0.092*	

### Atomic displacement parameters (Å^2^)

**Table:**

	*U*^11^	*U*^22^	*U*^33^	*U*^12^	*U*^13^	*U*^23^
Ru1	0.04018 (15)	0.03358 (14)	0.03059 (14)	−0.01658 (10)	−0.01405 (10)	0.00217 (9)
Cl1	0.0592 (5)	0.0374 (4)	0.0377 (4)	−0.0148 (3)	−0.0163 (3)	−0.0067 (3)
Cl2	0.0554 (5)	0.0370 (4)	0.0484 (4)	−0.0104 (3)	−0.0204 (4)	0.0055 (3)
Cl3	0.0483 (4)	0.0661 (5)	0.0460 (4)	−0.0327 (4)	−0.0158 (3)	0.0045 (4)
C1	0.0502 (19)	0.064 (2)	0.0352 (16)	−0.0304 (17)	−0.0113 (14)	0.0064 (15)
C2	0.0491 (18)	0.057 (2)	0.0325 (15)	−0.0231 (16)	−0.0090 (13)	−0.0027 (14)
C3	0.063 (2)	0.074 (2)	0.0285 (15)	−0.0371 (19)	−0.0185 (15)	0.0043 (15)
C4	0.059 (2)	0.072 (2)	0.0411 (17)	−0.0304 (19)	−0.0257 (16)	0.0210 (17)
C5	0.077 (3)	0.0430 (18)	0.0461 (19)	−0.0207 (17)	−0.0225 (18)	0.0171 (15)
C6	0.072 (2)	0.0484 (19)	0.0451 (18)	−0.0386 (18)	−0.0222 (17)	0.0124 (15)
C7	0.052 (2)	0.115 (4)	0.059 (2)	−0.040 (2)	−0.0186 (18)	−0.004 (2)
C8	0.075 (4)	0.359 (14)	0.081 (4)	−0.099 (6)	−0.003 (3)	−0.044 (6)
C9	0.079 (3)	0.136 (5)	0.076 (3)	−0.051 (3)	−0.040 (3)	−0.002 (3)
C10	0.072 (3)	0.131 (5)	0.073 (3)	−0.032 (3)	−0.046 (2)	0.026 (3)
S1	0.0542 (7)	0.0669 (8)	0.0728 (8)	−0.0065 (5)	−0.0317 (6)	−0.0088 (6)
C16'	0.0542 (7)	0.0669 (8)	0.0728 (8)	−0.0065 (5)	−0.0317 (6)	−0.0088 (6)
S2	0.1003 (11)	0.0355 (6)	0.0966 (11)	−0.0253 (6)	−0.0681 (9)	0.0179 (6)
C21'	0.1003 (11)	0.0355 (6)	0.0966 (11)	−0.0253 (6)	−0.0681 (9)	0.0179 (6)
N1	0.0445 (15)	0.0354 (14)	0.0491 (15)	−0.0070 (12)	−0.0116 (12)	−0.0012 (12)
N2	0.0473 (14)	0.0315 (13)	0.0384 (13)	−0.0116 (11)	−0.0141 (11)	0.0016 (10)
C11	0.0430 (16)	0.0340 (15)	0.0372 (15)	−0.0124 (13)	−0.0158 (13)	0.0010 (12)
C12	0.060 (2)	0.050 (2)	0.0482 (19)	−0.0202 (17)	−0.0080 (17)	0.0082 (16)
C13	0.060 (2)	0.051 (2)	0.059 (2)	−0.0159 (18)	−0.0004 (18)	−0.0002 (18)
C14	0.0436 (17)	0.0380 (17)	0.0561 (19)	0.0002 (14)	−0.0208 (15)	−0.0042 (15)
C15	0.0430 (17)	0.0376 (16)	0.0485 (18)	−0.0077 (13)	−0.0172 (14)	0.0023 (14)
S1'	0.092 (2)	0.0512 (17)	0.090 (2)	−0.0105 (15)	−0.0439 (19)	−0.0045 (15)
C16	0.092 (2)	0.0512 (17)	0.090 (2)	−0.0105 (15)	−0.0439 (19)	−0.0045 (15)
C17	0.097 (3)	0.053 (2)	0.068 (3)	−0.021 (2)	−0.023 (2)	−0.011 (2)
C18	0.095 (3)	0.075 (3)	0.070 (3)	−0.038 (3)	−0.044 (3)	0.005 (2)
C19	0.0448 (17)	0.0319 (15)	0.0511 (18)	−0.0088 (13)	−0.0242 (14)	−0.0002 (13)
C20	0.0374 (15)	0.0326 (15)	0.0533 (18)	−0.0059 (12)	−0.0197 (14)	−0.0032 (13)
S2'	0.0721 (15)	0.0671 (15)	0.0606 (13)	−0.0293 (11)	−0.0207 (11)	0.0003 (10)
C21	0.0721 (15)	0.0671 (15)	0.0606 (13)	−0.0293 (11)	−0.0207 (11)	0.0003 (10)
C22	0.060 (3)	0.115 (4)	0.082 (3)	−0.020 (3)	−0.015 (2)	−0.053 (3)
C23	0.067 (3)	0.0316 (18)	0.133 (5)	−0.0068 (18)	−0.041 (3)	−0.013 (2)

### Geometric parameters (Å, °)

**Table:**

Ru1—C6	2.140 (3)	C10—H10C	0.9599
Ru1—C2	2.154 (3)	S1—C15	1.700 (3)
Ru1—C5	2.173 (3)	S2—C20	1.647 (3)
Ru1—C1	2.180 (3)	N1—C11	1.307 (4)
Ru1—C3	2.191 (3)	N1—C13	1.458 (5)
Ru1—C4	2.207 (3)	N1—C14	1.462 (4)
Ru1—Cl1	2.4157 (11)	N2—C11	1.302 (4)
Ru1—Cl2	2.4329 (11)	N2—C19	1.460 (4)
Ru1—Cl3	2.4417 (11)	N2—C12	1.466 (4)
C1—C6	1.406 (5)	C11—H11	0.9600
C1—C2	1.435 (5)	C12—C13	1.534 (5)
C1—C7	1.521 (5)	C12—H12A	0.9600
C2—C3	1.398 (5)	C12—H12B	0.9600
C2—H2	0.9600	C13—H13A	0.9600
C3—C4	1.419 (6)	C13—H13B	0.9600
C3—H3	0.9600	C14—C15	1.505 (5)
C4—C5	1.422 (5)	C14—H14A	0.9600
C4—C10	1.495 (5)	C14—H14B	0.9600
C5—C6	1.421 (5)	C15—S1'	1.438 (4)
C5—H5	0.9600	S1'—H16	0.960 (3)
C6—H6	0.9600	C17—C18	1.338 (7)
C7—C9	1.492 (6)	C17—H17	0.9600
C7—C8	1.502 (7)	C18—H18	0.9600
C7—H7	0.9600	C19—C20	1.495 (4)
C8—H8A	0.9599	C19—H19A	0.9600
C8—H8B	0.9599	C19—H19B	0.9600
C8—H8C	0.9599	C20—S2'	1.558 (4)
C9—H9A	0.9599	S2'—H21	0.960 (2)
C9—H9B	0.9599	C22—C23	1.310 (7)
C9—H9C	0.9599	C22—H22	0.9600
C10—H10A	0.9599	C23—H23	0.9600
C10—H10B	0.9599		
			
C6—Ru1—C2	68.59 (14)	Ru1—C6—H6	128.9
C6—Ru1—C5	38.46 (15)	C9—C7—C8	113.3 (5)
C2—Ru1—C5	80.93 (15)	C9—C7—C1	113.7 (4)
C6—Ru1—C1	37.98 (14)	C8—C7—C1	109.7 (4)
C2—Ru1—C1	38.66 (13)	C9—C7—H7	106.5
C5—Ru1—C1	69.25 (15)	C8—C7—H7	106.5
C6—Ru1—C3	81.08 (14)	C1—C7—H7	106.5
C2—Ru1—C3	37.52 (13)	C7—C8—H8A	109.5
C5—Ru1—C3	68.20 (15)	C7—C8—H8B	109.5
C1—Ru1—C3	69.15 (13)	H8A—C8—H8B	109.5
C6—Ru1—C4	68.92 (14)	C7—C8—H8C	109.5
C2—Ru1—C4	68.06 (14)	H8A—C8—H8C	109.5
C5—Ru1—C4	37.88 (15)	H8B—C8—H8C	109.5
C1—Ru1—C4	81.91 (13)	C7—C9—H9A	109.5
C3—Ru1—C4	37.65 (15)	C7—C9—H9B	109.5
C6—Ru1—Cl1	86.77 (10)	H9A—C9—H9B	109.5
C2—Ru1—Cl1	144.83 (9)	C7—C9—H9C	109.5
C5—Ru1—Cl1	95.39 (11)	H9A—C9—H9C	109.5
C1—Ru1—Cl1	107.28 (10)	H9B—C9—H9C	109.5
C3—Ru1—Cl1	163.52 (11)	C4—C10—H10A	109.5
C4—Ru1—Cl1	126.94 (11)	C4—C10—H10B	109.5
C6—Ru1—Cl2	124.11 (11)	H10A—C10—H10B	109.5
C2—Ru1—Cl2	87.76 (11)	C4—C10—H10C	109.5
C5—Ru1—Cl2	162.10 (11)	H10A—C10—H10C	109.5
C1—Ru1—Cl2	93.29 (10)	H10B—C10—H10C	109.5
C3—Ru1—Cl2	110.15 (11)	C11—N1—C13	110.3 (3)
C4—Ru1—Cl2	146.86 (11)	C11—N1—C14	125.0 (3)
Cl1—Ru1—Cl2	85.88 (4)	C13—N1—C14	123.6 (3)
C6—Ru1—Cl3	147.20 (11)	C11—N2—C19	126.2 (3)
C2—Ru1—Cl3	126.23 (9)	C11—N2—C12	110.5 (3)
C5—Ru1—Cl3	110.17 (12)	C19—N2—C12	123.3 (3)
C1—Ru1—Cl3	164.68 (9)	N2—C11—N1	113.6 (3)
C3—Ru1—Cl3	96.15 (10)	N2—C11—H11	123.2
C4—Ru1—Cl3	88.90 (10)	N1—C11—H11	123.2
Cl1—Ru1—Cl3	88.04 (4)	N2—C12—C13	102.5 (3)
Cl2—Ru1—Cl3	87.71 (4)	N2—C12—H12A	111.3
C6—C1—C2	116.7 (3)	C13—C12—H12A	111.3
C6—C1—C7	124.6 (4)	N2—C12—H12B	111.3
C2—C1—C7	118.6 (4)	C13—C12—H12B	111.3
C6—C1—Ru1	69.44 (19)	H12A—C12—H12B	109.2
C2—C1—Ru1	69.67 (19)	N1—C13—C12	103.2 (3)
C7—C1—Ru1	128.6 (3)	N1—C13—H13A	111.1
C3—C2—C1	122.3 (3)	C12—C13—H13A	111.1
C3—C2—Ru1	72.69 (19)	N1—C13—H13B	111.1
C1—C2—Ru1	71.67 (19)	C12—C13—H13B	111.1
C3—C2—H2	118.9	H13A—C13—H13B	109.1
C1—C2—H2	118.9	N1—C14—C15	112.6 (3)
Ru1—C2—H2	129.3	N1—C14—H14A	109.1
C2—C3—C4	120.1 (3)	C15—C14—H14A	109.1
C2—C3—Ru1	69.79 (18)	N1—C14—H14B	109.1
C4—C3—Ru1	71.78 (19)	C15—C14—H14B	109.1
C2—C3—H3	119.9	H14A—C14—H14B	107.8
C4—C3—H3	119.9	S1'—C15—C14	126.7 (3)
Ru1—C3—H3	131.2	S1'—C15—S1	112.4 (3)
C3—C4—C5	118.9 (3)	C14—C15—S1	120.8 (2)
C3—C4—C10	120.4 (4)	C15—S1'—H16	126.4 (3)
C5—C4—C10	120.7 (4)	C18—C17—H17	122.6
C3—C4—Ru1	70.57 (19)	C17—C18—H18	123.4
C5—C4—Ru1	69.77 (19)	N2—C19—C20	112.8 (3)
C10—C4—Ru1	129.5 (3)	N2—C19—H19A	109.0
C6—C5—C4	119.9 (4)	C20—C19—H19A	109.0
C6—C5—Ru1	69.50 (19)	N2—C19—H19B	109.0
C4—C5—Ru1	72.4 (2)	C20—C19—H19B	109.0
C6—C5—H5	120.1	H19A—C19—H19B	107.8
C4—C5—H5	120.1	C19—C20—S2'	125.7 (3)
Ru1—C5—H5	130.7	C19—C20—S2	122.6 (3)
C1—C6—C5	122.1 (3)	S2'—C20—S2	111.7 (2)
C1—C6—Ru1	72.58 (19)	C20—S2'—H21	129.3 (2)
C5—C6—Ru1	72.0 (2)	C23—C22—H22	121.2
C1—C6—H6	119.0	C22—C23—H23	122.8
C5—C6—H6	119.0		
			
C2—Ru1—C1—C6	130.7 (3)	Cl2—Ru1—C4—C5	−150.0 (2)
C5—Ru1—C1—C6	28.9 (2)	Cl3—Ru1—C4—C5	125.9 (2)
C3—Ru1—C1—C6	102.6 (2)	C6—Ru1—C4—C10	−143.0 (5)
C4—Ru1—C1—C6	65.9 (2)	C2—Ru1—C4—C10	142.5 (5)
Cl1—Ru1—C1—C6	−60.4 (2)	C5—Ru1—C4—C10	−113.6 (5)
Cl2—Ru1—C1—C6	−147.07 (19)	C1—Ru1—C4—C10	180.0 (5)
Cl3—Ru1—C1—C6	119.6 (4)	C3—Ru1—C4—C10	113.8 (5)
C6—Ru1—C1—C2	−130.7 (3)	Cl1—Ru1—C4—C10	−74.4 (5)
C5—Ru1—C1—C2	−101.8 (2)	Cl2—Ru1—C4—C10	96.4 (4)
C3—Ru1—C1—C2	−28.1 (2)	Cl3—Ru1—C4—C10	12.3 (4)
C4—Ru1—C1—C2	−64.8 (2)	C3—C4—C5—C6	0.2 (5)
Cl1—Ru1—C1—C2	168.98 (18)	C10—C4—C5—C6	177.4 (3)
Cl2—Ru1—C1—C2	82.3 (2)	Ru1—C4—C5—C6	52.7 (3)
Cl3—Ru1—C1—C2	−11.0 (5)	C3—C4—C5—Ru1	−52.5 (3)
C6—Ru1—C1—C7	118.5 (4)	C10—C4—C5—Ru1	124.7 (3)
C2—Ru1—C1—C7	−110.9 (4)	C2—Ru1—C5—C6	−66.8 (2)
C5—Ru1—C1—C7	147.4 (4)	C1—Ru1—C5—C6	−28.6 (2)
C3—Ru1—C1—C7	−138.9 (4)	C3—Ru1—C5—C6	−103.6 (2)
C4—Ru1—C1—C7	−175.6 (4)	C4—Ru1—C5—C6	−132.6 (3)
Cl1—Ru1—C1—C7	58.1 (4)	Cl1—Ru1—C5—C6	77.9 (2)
Cl2—Ru1—C1—C7	−28.6 (4)	Cl2—Ru1—C5—C6	−15.4 (5)
Cl3—Ru1—C1—C7	−121.9 (4)	Cl3—Ru1—C5—C6	167.78 (19)
C6—C1—C2—C3	1.9 (5)	C6—Ru1—C5—C4	132.6 (3)
C7—C1—C2—C3	178.4 (3)	C2—Ru1—C5—C4	65.7 (2)
Ru1—C1—C2—C3	54.6 (3)	C1—Ru1—C5—C4	104.0 (2)
C6—C1—C2—Ru1	−52.7 (3)	C3—Ru1—C5—C4	29.0 (2)
C7—C1—C2—Ru1	123.8 (3)	Cl1—Ru1—C5—C4	−149.5 (2)
C6—Ru1—C2—C3	−103.7 (2)	Cl2—Ru1—C5—C4	117.2 (3)
C5—Ru1—C2—C3	−65.8 (2)	Cl3—Ru1—C5—C4	−59.6 (2)
C1—Ru1—C2—C3	−133.8 (3)	C2—C1—C6—C5	−1.9 (5)
C4—Ru1—C2—C3	−28.7 (2)	C7—C1—C6—C5	−178.1 (3)
Cl1—Ru1—C2—C3	−152.29 (18)	Ru1—C1—C6—C5	−54.7 (3)
Cl2—Ru1—C2—C3	128.1 (2)	C2—C1—C6—Ru1	52.8 (3)
Cl3—Ru1—C2—C3	42.6 (3)	C7—C1—C6—Ru1	−123.4 (3)
C6—Ru1—C2—C1	30.1 (2)	C4—C5—C6—C1	0.9 (5)
C5—Ru1—C2—C1	68.0 (2)	Ru1—C5—C6—C1	54.9 (3)
C3—Ru1—C2—C1	133.8 (3)	C4—C5—C6—Ru1	−54.0 (3)
C4—Ru1—C2—C1	105.1 (2)	C2—Ru1—C6—C1	−30.6 (2)
Cl1—Ru1—C2—C1	−18.5 (3)	C5—Ru1—C6—C1	−133.4 (3)
Cl2—Ru1—C2—C1	−98.1 (2)	C3—Ru1—C6—C1	−67.4 (2)
Cl3—Ru1—C2—C1	176.40 (17)	C4—Ru1—C6—C1	−104.4 (2)
C1—C2—C3—C4	−0.9 (5)	Cl1—Ru1—C6—C1	123.77 (19)
Ru1—C2—C3—C4	53.2 (3)	Cl2—Ru1—C6—C1	41.0 (2)
C1—C2—C3—Ru1	−54.1 (3)	Cl3—Ru1—C6—C1	−154.91 (17)
C6—Ru1—C3—C2	66.3 (2)	C2—Ru1—C6—C5	102.8 (2)
C5—Ru1—C3—C2	104.0 (2)	C1—Ru1—C6—C5	133.4 (3)
C1—Ru1—C3—C2	28.8 (2)	C3—Ru1—C6—C5	66.0 (2)
C4—Ru1—C3—C2	133.2 (3)	C4—Ru1—C6—C5	29.0 (2)
Cl1—Ru1—C3—C2	109.2 (4)	Cl1—Ru1—C6—C5	−102.8 (2)
Cl2—Ru1—C3—C2	−56.9 (2)	Cl2—Ru1—C6—C5	174.34 (18)
Cl3—Ru1—C3—C2	−146.7 (2)	Cl3—Ru1—C6—C5	−21.5 (3)
C6—Ru1—C3—C4	−66.9 (2)	C6—C1—C7—C9	15.6 (6)
C2—Ru1—C3—C4	−133.2 (3)	C2—C1—C7—C9	−160.6 (4)
C5—Ru1—C3—C4	−29.1 (2)	Ru1—C1—C7—C9	−74.7 (5)
C1—Ru1—C3—C4	−104.3 (2)	C6—C1—C7—C8	−112.5 (6)
Cl1—Ru1—C3—C4	−23.9 (5)	C2—C1—C7—C8	71.4 (6)
Cl2—Ru1—C3—C4	169.95 (18)	Ru1—C1—C7—C8	157.2 (5)
Cl3—Ru1—C3—C4	80.1 (2)	C19—N2—C11—N1	178.8 (3)
C2—C3—C4—C5	−0.2 (5)	C12—N2—C11—N1	0.4 (4)
Ru1—C3—C4—C5	52.1 (3)	C13—N1—C11—N2	0.0 (4)
C2—C3—C4—C10	−177.4 (3)	C14—N1—C11—N2	−168.6 (3)
Ru1—C3—C4—C10	−125.1 (3)	C11—N2—C12—C13	−0.6 (4)
C2—C3—C4—Ru1	−52.3 (3)	C19—N2—C12—C13	−179.1 (3)
C6—Ru1—C4—C3	103.2 (2)	C11—N1—C13—C12	−0.4 (4)
C2—Ru1—C4—C3	28.6 (2)	C14—N1—C13—C12	168.4 (3)
C5—Ru1—C4—C3	132.6 (3)	N2—C12—C13—N1	0.6 (4)
C1—Ru1—C4—C3	66.1 (2)	C11—N1—C14—C15	93.3 (4)
Cl1—Ru1—C4—C3	171.72 (16)	C13—N1—C14—C15	−73.9 (4)
Cl2—Ru1—C4—C3	−17.4 (3)	N1—C14—C15—S1'	143.0 (3)
Cl3—Ru1—C4—C3	−101.6 (2)	N1—C14—C15—S1	−40.9 (4)
C6—Ru1—C4—C5	−29.4 (2)	C11—N2—C19—C20	109.6 (4)
C2—Ru1—C4—C5	−103.9 (2)	C12—N2—C19—C20	−72.2 (4)
C1—Ru1—C4—C5	−66.4 (2)	N2—C19—C20—S2'	−84.8 (4)
C3—Ru1—C4—C5	−132.6 (3)	N2—C19—C20—S2	98.6 (3)
Cl1—Ru1—C4—C5	39.2 (3)		

### Hydrogen-bond geometry (Å, °)

**Table:**

*D*—H···*A*	*D*—H	H···*A*	*D*···*A*	*D*—H···*A*
C11—H11···Cl1^i^	0.96	2.62	3.450 (4)	144
C14—H14A···Cl1^i^	0.96	2.82	3.553 (4)	134
C19—H19A···Cl2^i^	0.96	2.81	3.671 (4)	150
C23—H23···Cl1^ii^	0.96	2.66	3.549 (4)	154
C14—H14B···Cg2^iii^	0.96	2.83	3.784 (5)	171
C19—H19B···Cl1	0.96	2.86	3.759 (5)	157

Symmetry codes: (i) −*x*, −*y*+1, −*z*+2; (ii) −*x*, −*y*+2, −*z*+2; (iii) −*x*+1, −*y*+1, −*z*+2.
